# Global efforts in conquering lung cancer in China

**DOI:** 10.1186/s40880-015-0034-6

**Published:** 2015-07-28

**Authors:** Li Yan, Li Xu

**Affiliations:** The US Chinese Anti-Cancer Association, Martinez, ‎CA‎ 94553 USA; Beijing Cancer Hospital and Institute, Peking University School of Oncology, Beijing, 100142 P. R. China; Jiangsu Hengrui Medicine Co., LTD, 778 Dong Fang Road, 12 F, Pudong, Shanghai, 200122 P. R. China

**Keywords:** Lung cancer, Targeted agents, Biomarker, Personalized medicine, Healthcare in China

## Abstract

Lung cancer, the most prevalent and deadly malignancy in the world, poses a particularly critical healthcare challenge to China due to the rapidly increasing new cases and the unique cancer genetics in Chinese patient population. Substantial progress has been made in molecular diagnosis and personalized treatment of the disease. The field is now moving towards multiple new directions to include (1) new generation of targeted agents such as epidermal growth factor receptor and anaplastic lymphoma kinase inhibitors to overcome resistance to their early generation counterparts; and (2) deeper understanding of tumor genetics of each individual patient and consequently the application of biomarkers to guide personalized treatment as well as novel drug development including combination therapy. The increasing capacity in innovative cancer drug research and development is supported by extensive collaboration within China and globally, and across academia and industry, to build up expertise and infrastructure in early-phase clinical testing of novel drugs. With these combined efforts, new and better medicines will be available for lung cancer patients in China in the near future.

Lung cancer, the most prevalent and deadly malignancy, accounts for a staggering 1.6 million new cases diagnosed every year and approximately 21% of cancer deaths to the global cancer burden [1]. In China, lung cancer has become a particularly challenging disease because of air pollution and smoking with an estimated 733,280 new cases every year and 671,625 cancer-related deaths [2–5]. In this special issue of the *Chinese Journal of Cancer* (*CJC*), we have organized a series of articles reviewing the global collaborative effort in improving lung cancer treatment in China.

Lung cancer treatment in China has shown substantial advancements in recent years exemplified by the integration of targeted therapies in standard of care. In the landmark Iressa Pan-ASia Study (IPASS) study, gefitinib (Iressa), a mutated epidermal growth factor receptor (*EGFR*) inhibitor, demonstrated superiority to carboplatin and paclitaxel in treating patients with lung cancer in East Asia [6]. A follow-up study confirmed that *EGFR* mutation is the strongest predictive biomarker for progression-free survival and tumor response to first-line gefitinib versus carboplatin and paclitaxel [7]. Similarly, crizotinib (Xalkori), an anaplastic lymphoma kinase (*ALK*) inhibitor, has shown superiority compared with chemotherapy in lung cancer patients with *ALK* translocation [8]. The high prevalence of activating *EGFR* mutations (32.7%) and *ALK* rearrangements (6.8%) among lung cancer patients in China has further expanded the use of these targeted agents [9]. However, the enthusiasm in using these targeted agents also brought up new challenges in clinical practice including the debate of whether such agents can and should be used in patients with wild-type targets as reviewed by Zhou et al. [10]. The review provides an important perspective in the context of relatively scarce healthcare resources and the heterogeneous diagnostic approaches in China.

Despite the encouraging clinical efficacy delivered by targeted agents, these monotherapies benefit only small subsets of patients with oncogene-driven tumors and, further, virtually all develop resistance and succumb to relapsed diseases [11]. Depending on the mechanisms of resistance, patients with relapsed diseases may further benefit from (1) new generation of targeted agents designed to address the resistance mechanisms; and (2) combination therapy to target multiple pathways simultaneously including resistance mechanisms.

The article by Xu [12] describes the discovery and development of avitinib, an investigational third-generation EGFR inhibitor originally discovered in China, and is being developed in China and the United States (US). Similar to other third-generation EGFR inhibitors such as AZD9291 and CO1686, avitinib irreversibly inhibits the mutant *EGFR*, spares the wild-type *EGFR* thus avoiding or minimizing normal tissue toxicities, and overcomes T790M mutation-mediated resistance to early-generation EGFR inhibitors. The ongoing phase 1 study of avitinib in China in lung cancer patients with T790M mutation will provide the first set of clinical data of third-generation EGFR inhibitors in Chinese patients. The parallel clinical development in the US as an integrated global development strategy will generate data in relevant patient population in which other third-generation EGFR inhibitors are being investigated. It is highly expected that avitinib, an innovative drug discovered in China, has the potential to become the first-in-class new generation EGFR inhibitor in China.

The successful clinical approach to effectively treating lung cancer and addressing drug-resistance relies on the use of biomarkers to personalize cancer treatment. The review article by Fang et al. [13] from MD Anderson Cancer Center discusses predictive biomarkers not only for traditional targeted agents such as inhibitors of EGFR, ALK, and c-ros oncogene 1 receptor tyrosine kinase (ROS1) but also for immune checkpoint inhibitors. The article also highlights the need for identifying predictive biomarkers in anticancer drug development. To succeed in such biomarker-based drug development approach, a mature network of clinical trial sites with sound translational medicine capability is a prerequisite. Yang et al. [14] analyzed early-phase clinical trials in lung cancer in the US and conducted a comparison with the trials conducted by the Chinese Thoracic Oncology Group (CTONG), the first and largest thoracic oncology corporative group in China. The high percentage of early-phase clinical trials in the US provides a solid foundation for identifying, developing, and validating biomarkers to expedite drug development in late phases. In contrast, CTONG has yet to conduct a single early-phase clinical trial since its inception in 2007 at the time of the analysis. Part of the reasons for this discrepancy between the US and China resides in the lack of clinical trial staff, e.g., physicians and nurses, with experience in early-phase oncology trials. To address this gap, the US Chinese Anti-Cancer Association (USCACA), with generous support from Jiangsu Hengrui Medicine Co., LTD, has initiated the Early Phase Oncology Research Training (EFFORT) Program [15]. The scholarship will support training of young investigators from China in collaboration with US cancer centers and National Cancer Institute. Through short rotation programs, Chinese young investigators will gain experience in designing and conducting early-phase oncology clinical trials and bring the best practice to their home institutes in China [14].

We firmly believe that the advancement in our understanding in genetics underlying lung cancer, the rapidly growing innovative drug research and development (R&D) capacity, the enhanced global collaboration, and the expanding capability in cancer drug clinical development will lead to the next wave of breakthroughs in the treatment of lung cancer in a personalized fashion in China.
